# Fourteen-Year Follow-Up of a Patient With a Hydroxyapatite Ceramic Matrix Reconstruction of and a Bone Graft for a Critical-Size Cortical Bone Defect: A Case Report

**DOI:** 10.7759/cureus.63901

**Published:** 2024-07-05

**Authors:** Jai Thilak, Adarsh Venugopal, Venugopal Menon

**Affiliations:** 1 Orthopaedic Surgery, Amrita Hospital, Kochi, Kochi, IND; 2 Orthopaedic Surgery, Rajagiri Hospital, Kochi, IND

**Keywords:** ceramic, bone graft, hydroxyapetite, cortical bone substitute, chondrosarcoma

## Abstract

A 42-year-old man diagnosed with chondrosarcoma of the proximal femur underwent limb salvage by compartmental excision of the lesion and reconstruction with a custom-made hip prosthesis. The critical-size defect in the proximal femur was reconstructed with ceramic hemicylinders that were tied in place with sutures and augmented with two fibular strut grafts and an autologous cancellous iliac crest bone graft. A fourteen-year follow-up of the same case revealed that substituted ceramic matrices can be converted into dynamic, metabolically active, living bone.

## Introduction

Apart from cancellous bone augmentation in bony cavity filling, flat bone reconstruction, periodontal applications, etc., the use of synthetic materials in cortical bone defects of critical size has been limited [1]. Marcacci et al.'s study [2] of ceramic scaffolds seeded with autologous stem cells and Arai et al.'s study of fibular graft site reconstruction with ceramic matrix are the only reported good outcome studies [3]. The current study reports the long-term clinical, radiological, and histological outcomes of a critical-size cortical bone defect in a human reconstructed by a tissue engineering protocol.

Cancellous bone augmentation has been the first clinically established form of engineered bone usage. Injectable or putty-like matrices as well as granular or spherical shapes have been used to fill cavities and support cancellous bone defects. Limb osteotomies have been augmented with ceramic wedges [4,5], and spinal fusions have been performed with ceramic-augmented cages [6]. Cellular elements and growth factors have been added to enhance the biological activity of the augment [2]. But cancellous bone augmentation is the low-hanging fruit; the real challenge is cortical bone defects of the critical size that have been lost due to trauma, tumor resection, congenital defects, or infections [4].

A critical size bone defect is a rather loosely defined term to indicate the potential for non-union and the need for secondary surgical procedures. Various authors have defined it as “half the cortical diameter” and “2.5 cm” [6,7]. Conventional strategies employed for critical cortical defects are autologous fibular graft, cadaveric allograft, distraction osteogenesis [6,7], etc., and the induced membrane technique of Masquelet [8]. Each has its own set of advantages and disadvantages. Limited pilot studies have been performed with synthetic ceramic or polymer composites for critical bone defect reconstruction [9]. While the outcomes of such studies are largely measured by clinical and radiological means, tissue (histological) sampling in humans is not easy to perform for obvious ethical reasons. This study reports a case where such sampling was possible along with clinical-radiological evaluation. The patient was informed that data concerning the case would be submitted for publication and he agreed to the same. The case was approved by the Amrita Hospital Ethics Committee for the trial of implantation and was deemed inappropriate for conventional defect reconstruction procedures.

## Case presentation

**Figure 1:**
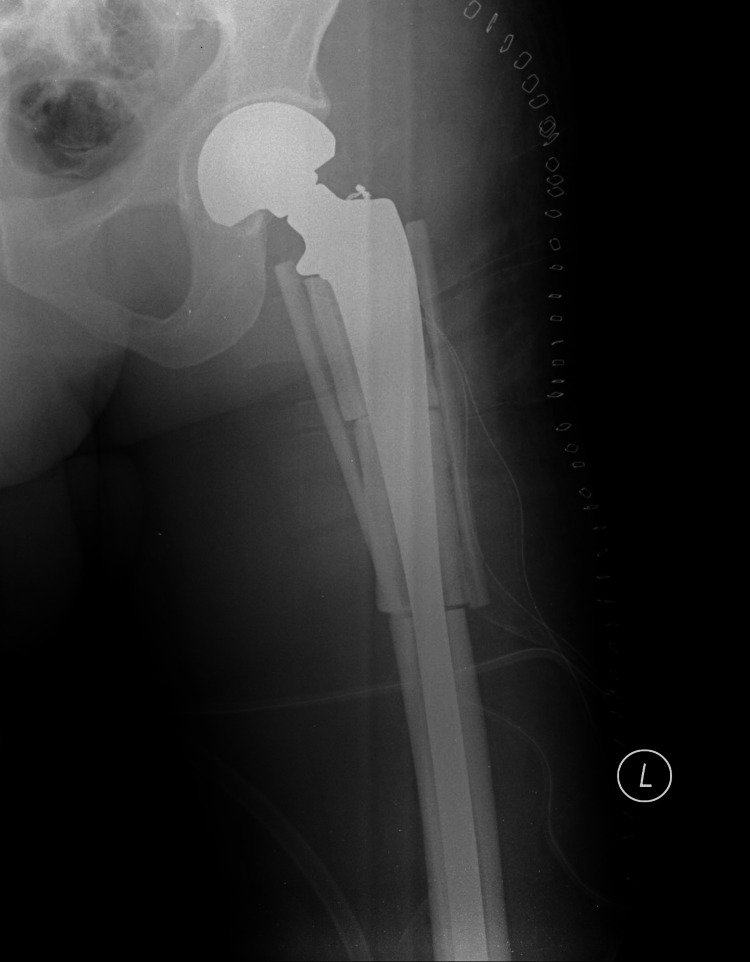
Day-one postoperative radiograph

We present the case of a male patient in his early 40s who was diagnosed with chondrosarcoma of the proximal femur and underwent a limb salvage procedure consisting of compartmental excision of the lesion and reconstruction with a custom-made hip prosthesis. The critical size defect in the proximal femur of 15 cm was reconstructed with ceramic hemicylinders that were fixed in place with sutures and augmented with two fibular strut grafts and an autologous cancellous iliac crest bone graft (Figure 1).

**Figure 2:**
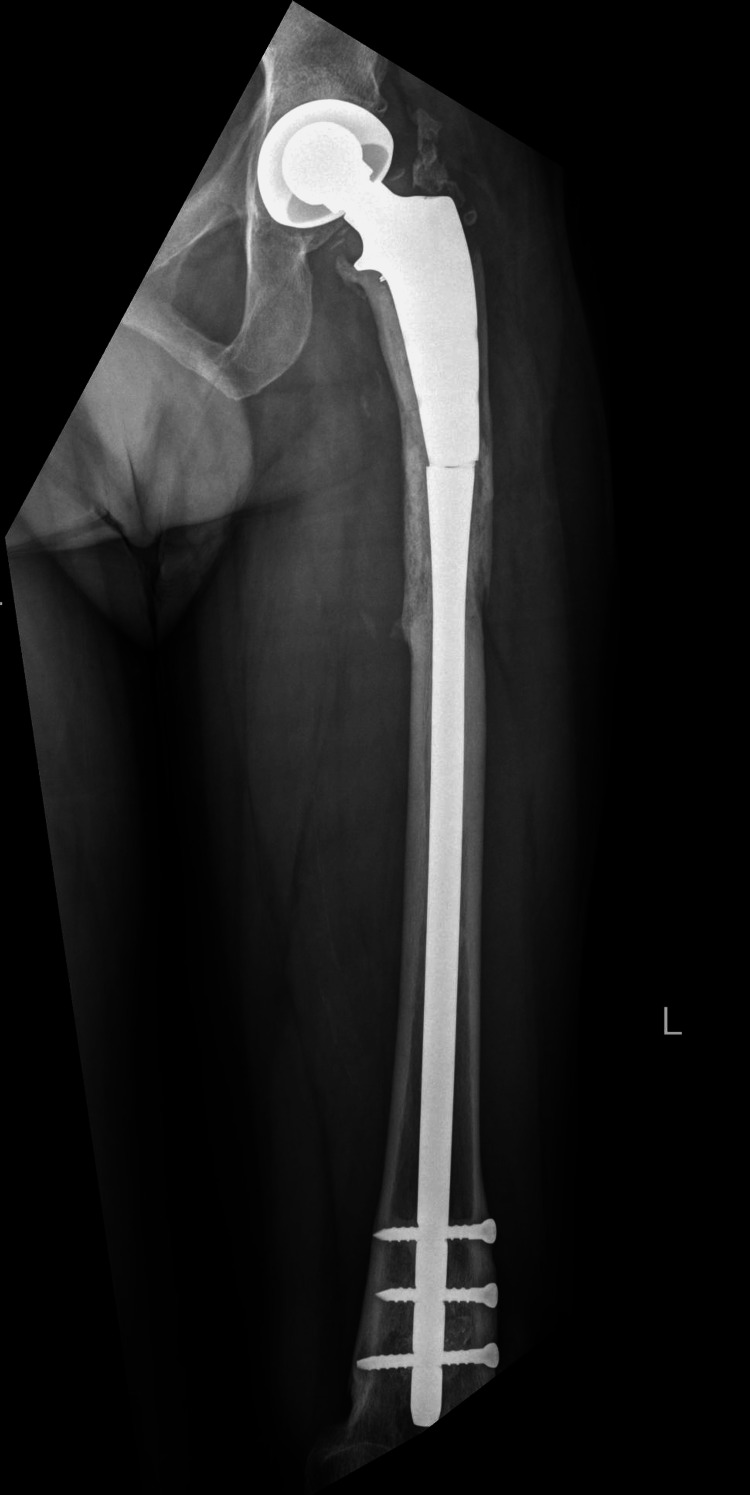
Fourteen years after index surgery radiograph showing implant failure

Postoperatively the patient was made to walk non-weight bearing in the operated limb initially and was asymptomatic till 14 years after the index surgery when he developed implant failure (Figure 2).

**Figure 3:**
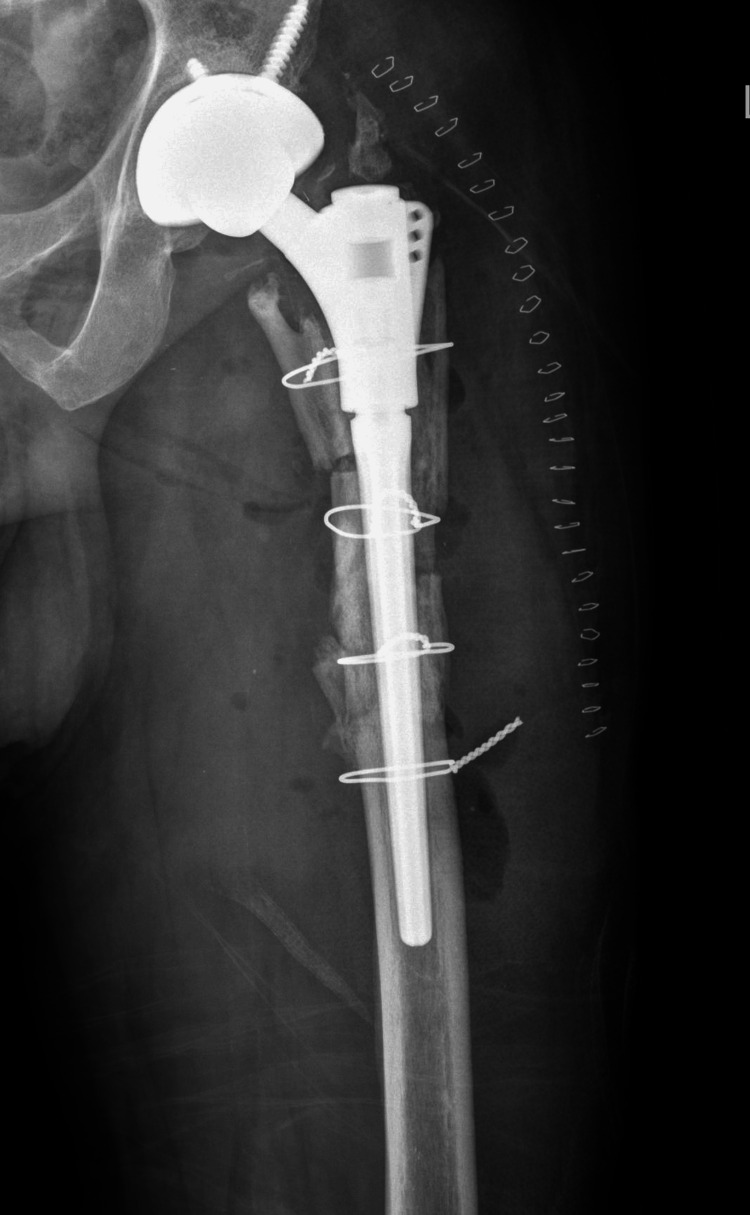
Immediate postoperative radiograph after revision total hip replacement (THR)

The custom tumour prosthesis was revised to uncemented total hip replacement (THR) with a distal loading stem augmented with cerclage wires (Figure 3).

Biopsy was obtained from the proximal edge of the femur (from the originally ceramic reconstructed area). His postoperative period was uneventful and he was discharged home with partial weight bearing for six weeks and to full weight bearing thereafter.

The ceramic matrix is an indigenously produced hydroxyapatite (HA)-bioactive glass composite of 80-20 ratio. The HA is a heat-sintered ceramic while the bio glass is a sol-gel derived low-temperature product. The ceramic hemicylinders were loaded with autologous bone marrow aspirate before implantation to provide cellular and growth factors into the surgical milieu. 

Salient radiological findings

**Figure 4:**
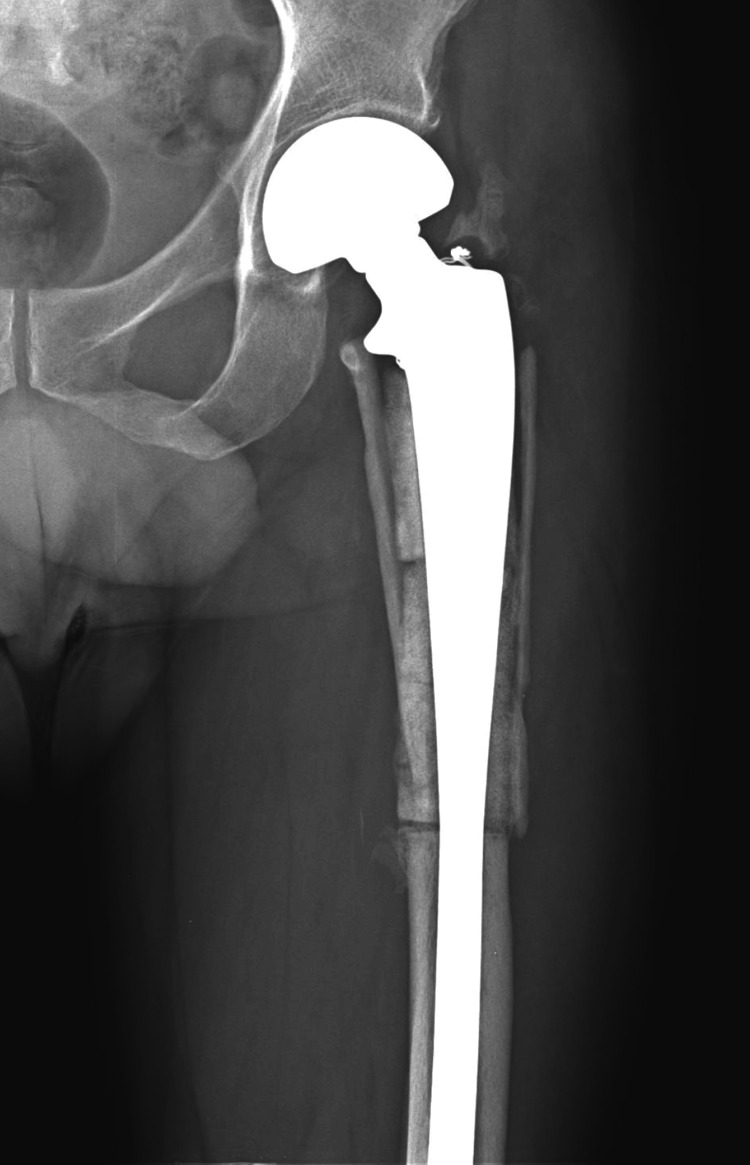
The radiodensity of the ceramic in a radiograph at one year

The radiodensity of the ceramic progressively diminished and became iso-dense with the normal bone (Figure 4).

The ceramic-ceramic slits (where hemicylinders meet) eventually filled out and became continuous. The fibular strut-ceramic interface remained open even in the 14-year-old X-ray suggesting that bone ingrowth in this region was incomplete. However, there were several regions where it did become continuous. The postoperative (revision) images show that some of the ceramic-ceramic interfaces were still soft and appeared to have de-bonded (Figure 3).

Salient histological findings

**Figure 5:**
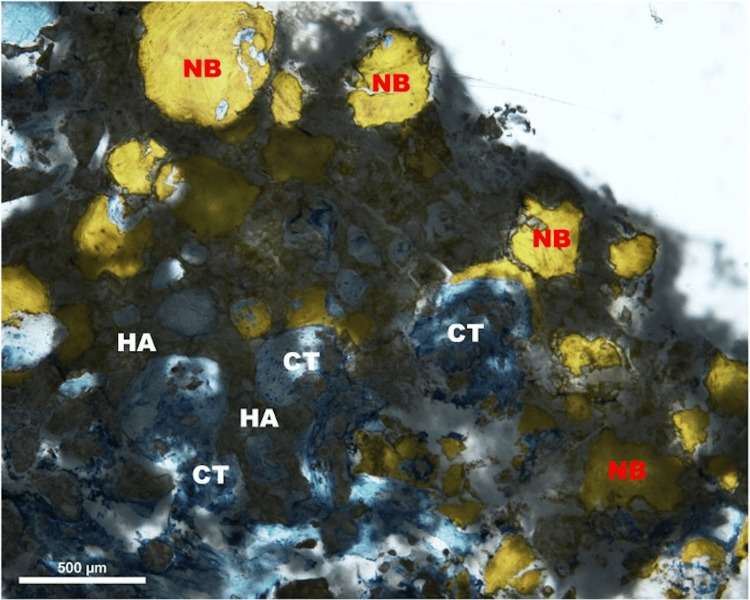
Light micrograph of the bone ceramic interface; Stevenel’s blue stain, 40x magnification. Newly formed woven bone (NB) formation with soft connective tissue (CT) proliferation and the presence of resorbing material graft (hydroxyapatite (HA))

Areas/islands of ceramic persisted even after 14 years of implantation (Figure 5).

**Figure 6:**
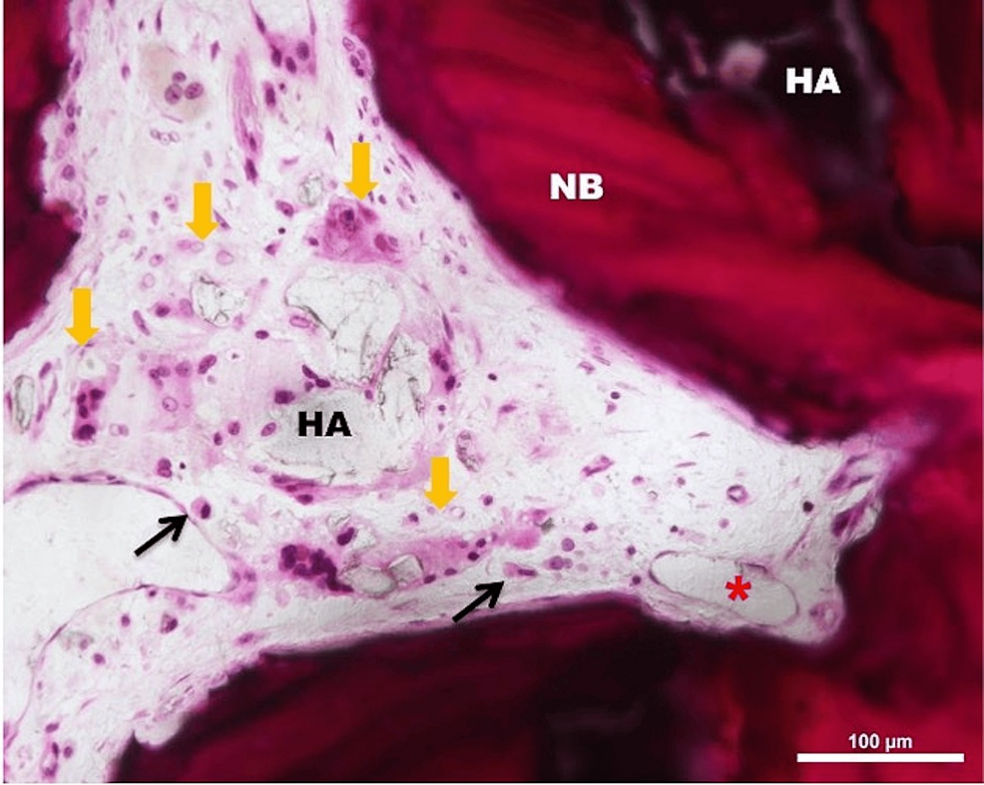
Light micrograph of the resorbing hydroxyapatite (HA) and newly formed woven bone (NB) area; H&E stain, 200x magnification. Graft material resorption by laden foreign body type giant cells (yellow arrows) with engulfed materials in their cytoplasm, macrophages (black arrow), and blood capillaries (asterisk)

The bone formed by replacing the ceramic is identical to normal mature cortical bone with Haversian systems and trabecular structure (Figure 6).

**Figure 7:**
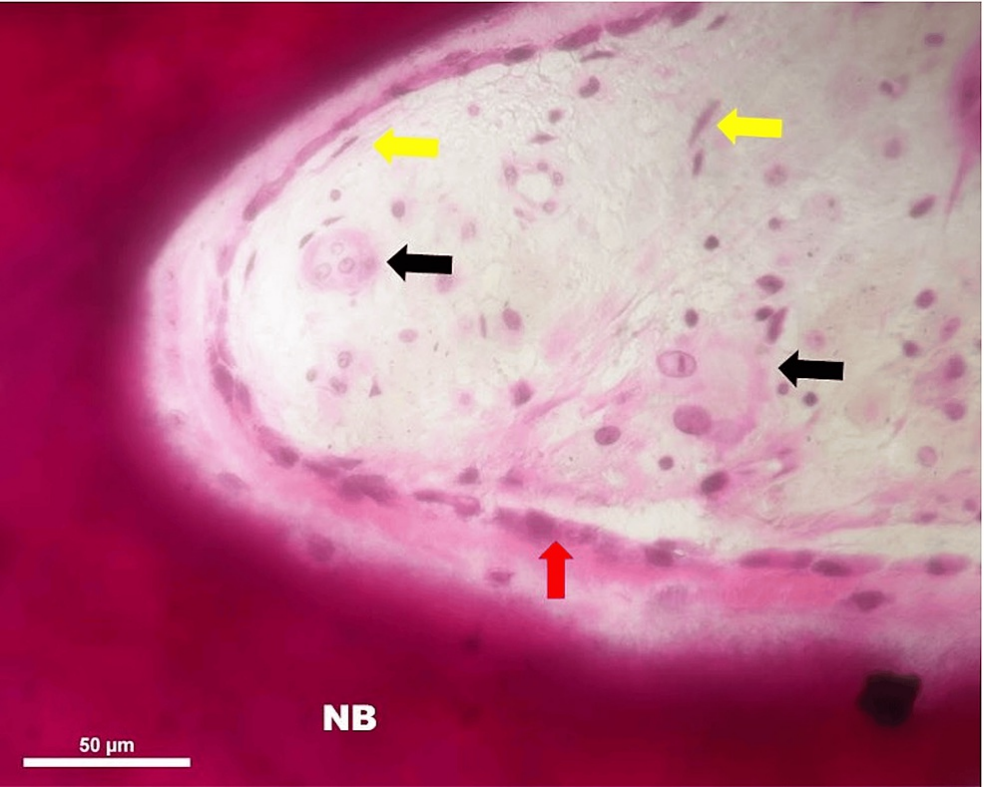
Light micrograph of the newly formed woven bone (NB); H&E stain, 400x magnification. NB with rosette like appearance of lining osteoblast cells (red arrow), multinucleated osteoclast giant cells (black arrow), and fibrocytes (yellow arrow)

The porosities and interstices were filled with connective tissue and vascular marrow-like structures (Figure 7).

**Figure 8:**
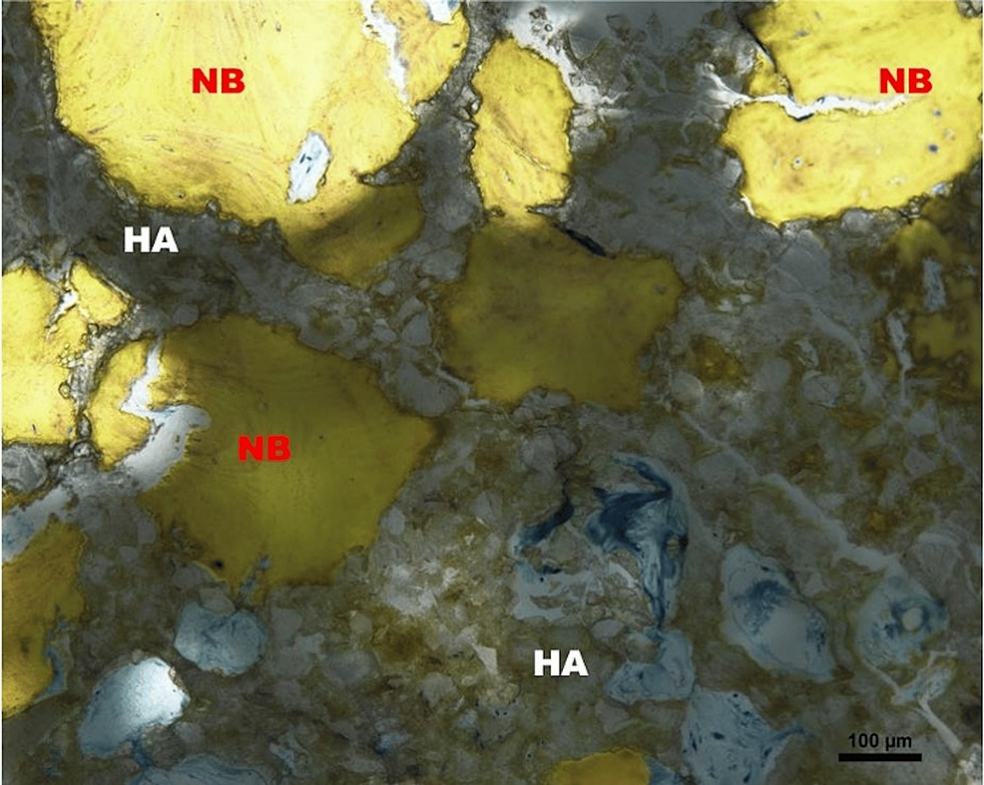
Light micrograph of the bone ceramic interface; Stevenel’s blue stain, 100x magnification. Newly formed woven bone (NB) formation with the presence of resorbing material graft (hydroxyapatite (HA))

The histology of the second case from the central cylinder showed no signs of infection but definite evidence of living lamellar bone ingrowth into the interstices and pores of the ceramic (Figure 8).

## Discussion

Critical size defects in bone are defined as gaps in cortical bone that will not heal with conservative management irrespective of the time passed [10]. Several authors have summarized the advances and existing methodologies to treat these difficult lesions [11,12]. The older techniques of vascularised and nonvascularized fibular grafting and distraction osteogenesis gave way to the newer techniques of Masquelet and RIA (reamer/irrigator/aspirator), each with its advantages and disadvantages [8,13-15]. Nevertheless, it is interesting to note that 36-40% of allografts needed removal between five and 10 years from implantation [16]. Kobbe’s case is technically similar to ours in that the autograft was membrane-guided over a metallic implant [17]. The concept of ceramic sleeves over metallic devices is not new; Huckstep in 1987 published his first report on this idea [18]. In the future, ceramic 3D printing will offer several advantages in terms of custom scaffold building, which can potentially revolutionize hard tissue engineering [18,19].

The concept of contact guidance based on the osteoconductive properties of ceramic to direct new bone formation [20] is well established. However, critical-sized cortical defects in humans treated with ceramic or other engineered materials have been limited. Roffi et al. in 2017 [6] published a systematic review of all the pre-clinical and clinical studies to date. Several animal studies using ceramic cylinders with or without cells and growth factors have reported excellent results [3]. There are four studies on human bones: three of them using HA and one tricalcium phosphate (TCP) (2,4-6). Two of them had augmented their ceramic matrix with autologous bone marrow-derived stromal cells (BMSCs). The follow-up duration was four months to seven years and the measured outcomes were the radiological integration of the ceramic and functional outcomes. The longest follow-up was that of Marcacci et al. [2] where four cases of long bone defects were filled with ceramic cylinders and stem cells and followed up for seven years with good radiological results. Arai et al.'s study [3] where fibular harvest sites were filled with ßTCP scaffolds in 14 patients concluded that fibular regeneration is better in children than adults. Both the above studies have relied on radiological follow-ups which is, for obvious reasons, the only practical possibility since none of them reported metallic device failure. We utilized this reported case of implant failure necessitating revision surgery as an opportunity to histologically sample the engineered tissue. 

This case demonstrates that the substituted ceramic matrix can be converted into dynamic, metabolically active, living bone. Lamellar bone formation along with Haversian system arrangement is seen in this new bone. It is capable of load-bearing as in normal bone since the prosthesis withstood full weight-bearing ambulation as in normal individuals. The newly formed bone also follows Pauwels’s laws of hypertrophy along lines of stress as demonstrated by the pedestal sign formed at the medial calcar region [16]. It is also evident that the newly formed bone can cross gaps in the ceramic (like the ceramic-ceramic edge and the cortical bone to the ceramic gap) and lay down new bone in the interstices as is seen radiologically [2]. 

 It is interesting to postulate the source of the new bone within the ceramic matrix. Some BMSCs would be viable within the substrate along with the growth factors provided by the bone marrow injections and the local tissue bleeding following surgery. Some of the live cells would have come from the surrounding cancellous bone graft as well. It is unlikely that the major source of living bone was the cut ends of the femur since the surface area is rather small and a small callus is visible on sequential X-rays.

## Conclusions

The case under study is an example of how substituted ceramic matrices can be converted into dynamic, metabolically active, living bone. The newly formed bone can cross gaps and lay down more new bone in the interstices. Moreover, the newly formed bone allows load-bearing like normal bone. The newly formed bone also follows Pauwels's law of hypertrophy along lines of stress.

## References

[1] de Boer HH (1988). The history of bone grafts. Clin Orthop Relat Res.

[2] Marcacci M, Kon E, Moukhachev V (2007). Stem cells associated with macroporous bioceramics for long bone repair: 6- to 7-year outcome of a pilot clinical study. Tissue Eng.

[3] Arai E, Nakashima H, Tsukushi S (2005). Regenerating the fibula with beta-tricalcium phosphate minimizes morbidity after fibula resection. Clin Orthop Relat Res.

[4] Bi L, Zobell B, Liu X, Rahaman MN, Bonewald LF (2014). Healing of critical-size segmental defects in rat femora using strong porous bioactive glass scaffolds. Mater Sci Eng C Mater Biol Appl.

[5] Roberts TT, Rosenbaum AJ (2012). Bone grafts, bone substitutes and orthobiologics: the bridge between basic science and clinical advancements in fracture healing. Organogenesis.

[6] Roffi A, Krishnakumar GS, Gostynska N, Kon E, Candrian C, Filardo G (2017). The role of three-dimensional scaffolds in treating long bone defects: evidence from preclinical and clinical literature-a systematic review. Biomed Res Int.

[7] Schemitsch EH (2017). Size matters: defining critical in bone defect size!. J Orthop Trauma.

[8] Masquelet AC, Begue T (2010). The concept of induced membrane for reconstruction of long bone defects. Orthop Clin North Am.

[9] Azi ML, Teixeira AA, Cotias RB, Joeris A, Kfuri M (2019). Induced-membrane technique in the management of posttraumatic bone defects. JBJS Essent Surg Tech.

[10] Fiume E, Migneco C, Verné E, Baino F (2020). Comparison between bioactive sol-gel and melt-derived glasses/glass-ceramics based on the multicomponent SiO(2)-P(2)O(5)-CaO-MgO-Na(2)O-K(2)O system. Materials (Basel).

[11] Roddy E, DeBaun MR, Daoud-Gray A, Yang YP, Gardner MJ (2018). Treatment of critical-sized bone defects: clinical and tissue engineering perspectives. Eur J Orthop Surg Traumatol.

[12] Dimitriou R, Jones E, McGonagle D, Giannoudis PV (2011). Bone regeneration: current concepts and future directions. BMC Med.

[13] Nade S (2002). The replacement of broken, missing and diseased bone. Bone in Clinical Orthopaedics.

[14] Giannoudis PV, Harwood PJ, Tosounidis T, Kanakaris NK (2016). Restoration of long bone defects treated with the induced membrane technique: protocol and outcomes. Injury.

[15] Le Baron M, Vivona JP, Maman P, Volpi R, Flecher X (2019). Can the reamer/irrigator/aspirator system replace anterior iliac crest grafting when treating long bone nonunion?. Orthop Traumatol Surg Res.

[16] Aponte-Tinao LA, Ayerza MA, Albergo JI, Farfalli GL (2020). Do massive allograft reconstructions for tumors of the femur and tibia survive 10 or more years after implantation?. Clin Orthop Relat Res.

[17] Kobbe P, Laubach M, Hutmacher DW, Alabdulrahman H, Sellei RM, Hildebrand F (2020). Convergence of scaffold-guided bone regeneration and RIA bone grafting for the treatment of a critical-sized bone defect of the femoral shaft. Eur J Med Res.

[18] Huckstep RL (1987). Stabilization and prosthetic replacement in difficult fractures and bone tumors. Clin Orthop Relat Res.

[19] Feng Y, Zhu S, Mei D, Li J, Zhang J, Yang S, Guan S (2021). Application of 3D printing technology in bone tissue engineering: a review. Curr Drug Deliv.

[20] Gwon Y, Park S, Kim W, Han T, Kim H, Kim J (2021). Radially patterned transplantable biodegradable scaffolds as topographically defined contact guidance platforms for accelerating bone regeneration. J Biol Eng.

